# Multi-Omics Evidence Linking Depression to MASLD Risk via Inflammatory Immune Signaling

**DOI:** 10.3390/biomedicines14010174

**Published:** 2026-01-13

**Authors:** Keye Lin, Yiwei Liu, Xitong Liang, Yiming Zhang, Zijie Luo, Fei Chen, Runhua Zhang, Peiyu Ma, Xiang Chen

**Affiliations:** 1 Department of Clinical Medicine, The First School of Clinical Medicine, Guangzhou Medical University, Guangzhou 511436, China; oldhorse041007@163.com (K.L.);; 2 Department of Clinical Medicine, Nanshan School, Guangzhou Medical University, Guangzhou 511436, China; 3 Department of Neurology, The First Affiliated Hospital of Guangzhou Medical University, Guangzhou 510120, China

**Keywords:** depression, MASLD, inflammation, *CD40LG*, immune signaling, sex differences

## Abstract

**Background**: Depression and Metabolic Dysfunction-Associated Steatotic Fatty Liver Disease (MASLD) are common chronic diseases, respectively. However, the causal and molecular links between them remain unclear. In order to explore whether depression contributes to an increased risk of MASLD and whether inflammation mediates this effect, we integrated multi-level evidence from the epidemiology of the National Health and Nutrition Examination Survey (NHANES), the genetics of GWAS, the transcriptomes of GEO, and single-cell RNA sequencing datasets. **Methods**: A multi-level integrative analysis strategy was used to validate this pathway. First, a cross-sectional epidemiological analysis based on NHANES data was used to reveal the association between depression and MASLD, and to explore the mediating role of inflammation and liver injury markers. Secondly, a two-sample Mendelian randomization analysis was used to infer the causal direction of depression and MASLD, and to verify the mediating effect of systemic inflammation and liver injury indicators at the genetic level. Then, the transcriptome co-expression network analysis and machine learning were used to screen the common hub genes connecting the two diseases. Finally, single-cell transcriptome data were used to characterize the dynamic expression of potential key genes during disease progression at cellular resolution. **Results**: Depression significantly increased the risk of MASLD, especially in women (OR = 1.39, 95%CI [1.17–1.65]). Parallel mediation analysis showed that high-sensitivity C-reactive protein (hs-CRP) (*p* < 0.001), γ-glutamyltransferase (GGT) (*p* < 0.001), and alkaline phosphatase (ALP) (*p* < 0.001) mediated this relationship. Mendelian randomization analysis confirmed the unidirectional causal effect of depression on MASLD, and there was no reverse association (β = 0.483, SE = 0.146, *p* = 0.001). Weighted gene co-expression network analysis and machine learning identified *CD40LG* as a potential molecular bridge between depression-associated immune modules and MASLD. In addition, single-cell data analysis revealed a stage-specific trend of *CD40LG* expression in *CD4*^+^ T cells during MASLD progression, while its receptor *CD40* was also activated in B cells. In the female sample, *CD40LG* maintained an upward trend. However, the stability of this result is limited by the limited sample size. **Conclusions**: This study provides converging multi-omics evidence that depression plays a causal role in MASLD through inflammation-mediated immune signaling. The *CD40LG*-*CD40* axis has emerged as an immune mechanism that transposes depression into the pathogenesis of MASLD, providing a potential target for the intervention of gender-specific metabolic liver disease.

## 1. Introduction

Depression is one of the most prevalent mental health disorders worldwide. According to the 2021 Global Burden of Disease (GBD) study, depression affects over 330 million people and accounts for more than 56 million disability-adjusted life years (DALYs), with consistently higher prevalence in females than in males [1]. Metabolic dysfunction–associated steatotic liver disease (MASLD), formerly known as nonalcoholic fatty liver disease (NAFLD), is the most common chronic liver disease globally. It affects over one-third of the adult population and contributes to more than 4 million DALYs [2,3]. Like depression, MASLD is a chronic, progressive condition whose prevalence has steadily risen over the past three decades. Notably, a recent study found that 26.3% of MASLD patients also have depression, indicating a substantial overlap between these conditions [4].

Accumulating evidence suggests that chronic, low-grade inflammation serves as a common pathophysiological platform linking depression and MASLD. Recent reviews emphasize that systemic inflammation is a key modifiable contributor to depression, with elevated cytokines and acute-phase proteins being hallmarks of this state [5]. On the other hand, MASLD is intrinsically linked to hepatic and systemic inflammation, and research by A. Teixeira et al. identified the Systemic Immune-Inflammation Index (SII) as a reliable and independent risk marker for the disease [6]. This forms a cross-organ inflammatory network that links central neuroinflammation to hepatic metabolic dysfunction. The research by E. F. Osimo et al. confirmed that depression is associated with significantly increased levels of multiple inflammatory markers, including CRP, IL-6, and TNF-α. Notably, certain immune markers—such as CRP, IL-12, and sIL-2R—exhibit reduced variability in depressed patients, suggesting a more homogeneous inflammatory phenotype in this population [7]. Furthermore, approximately 20–30% of patients with depression fall into the category of “immuno-metabolic depression,” characterized by atypical energy-related symptoms, systemic low-grade inflammation, and metabolic abnormalities that include obesity, dyslipidemia, insulin and leptin resistance—features commonly associated with MASLD [8]. Nevertheless, the causal relationship between depression and MASLD remains controversial.

Although some studies have linked depression and MASLD through inflammation, the causal relationship is still controversial. Mendelian randomization (MR) studies, which use genetic instruments to mitigate confounding, have produced inconsistent results. For example, Lv et al. reported a significant causal effect of depression on MASLD risk [9], whereas Li et al. found no causal relationship [10].

The aforementioned evidence suggests that depression may promote the occurrence of MASLD through inflammation-centered immune dysregulation. To systematically validate this pathway, this study employs a multi-level integrated analytical strategy: first, cross-sectional epidemiological analysis based on National Health and Nutrition Examination Survey (NHANES) data is used to reveal the association between depression and MASLD and preliminarily screen the mediating effects of inflammatory and liver injury markers; second, two-sample Mendelian randomization analysis is applied to infer the causal direction between depression and MASLD and to validate the mediating effects of systemic inflammation (e.g., CRP) and liver enzymes (e.g., GGT, ALP) at the genetic level; next, transcriptomic co-expression network analysis combined with machine learning is conducted to identify shared immune-inflammatory molecular modules and hub genes linking depression and MASLD; finally, single-cell transcriptomic data are utilized to validate the dynamic expression of key molecules during disease progression at cellular resolution. This research framework, integrating macro-level associations, causal inference, and molecular mechanisms, aims to provide convergent evidence for the “depression–inflammation–MASLD” pathway and uncover the potential sex-specific immune mechanisms underlying it.

## 2. Materials and Methods

### 2.1. Study Design and Data Sources

A multilevel integrative design was employed, combining four complementary evidence streams: (i) a cross-sectional epidemiological analysis of depressive symptoms and MASLD using NHANES 2017–2020 data; (ii) a two-sample Mendelian randomization (MR) analysis to infer causality; (iii) transcriptome-wide co-expression network analysis combined with supervised machine learning to identify shared molecular mechanisms; and (iv) single-cell analyses to examine transcriptomic mechanisms at cellular resolution.

### 2.2. NHANES Disease Definitions and Analytic Strategy

NHANES 2017–2020 data were used for the cross-sectional analysis; the sample composition is shown in Table 1. MASLD was defined according to the 2023 international consensus, which requires evidence of hepatic steatosis—assessed by a controlled attenuation parameter (CAP) threshold—and at least one cardiometabolic risk factor: body-mass index (BMI) ≥ 25 kg/m^2^ or central obesity; impaired fasting glucose or hemoglobin A1c (HbA1c) ≥ 5.7% or diagnosed diabetes; elevated blood pressure or use of antihypertensive treatment; triglycerides ≥150 mg/dL or lipid-lowering therapy; or low high-density lipoprotein cholesterol (HDL-C). Participants with viral hepatitis or excess alcohol intake (>20 g/day in women; >30 g/day in men) were excluded. Depressive symptoms were assessed with the 9-item Patient Health Questionnaire (PHQ-9). The primary definition of depressive symptoms was PHQ-9 > 4; sensitivity analyses used thresholds of ≥10, ≥15, and ≥20, and PHQ-9 was also analyzed as a continuous measure [11].

Multivariable logistic regression models were constructed to examine the association between depression and MASLD. Models were estimated in the overall sample, adjusting for age and sex, followed by sex-stratified analyses. Effect modification by sex was formally evaluated using an interaction model including terms for depression, sex, their interaction, and age; the interaction coefficient and its 95% confidence interval (CI) were reported.

### 2.3. Mendelian Randomization

Two-sample MR was conducted to infer the causal relationship between depression and MASLD. Genetic instruments for depression were drawn from a meta-analysis by Howard et al., comprising 807,553 individuals of European ancestry (246,363 cases and 561,190 controls) [12]. Summary statistics for non-alcoholic fatty liver disease (NAFLD, used as a proxy for MASLD) were obtained from the FinnGen consortium (R10: 426,641 participants with 2568 cases; R12: 516,561 participants with 3649 cases) [13].

To evaluate mediation of the depression on MASLD’s effect by candidate biomarkers identified in the cross-sectional analysis, single-mediator and parallel-mediator analyses were performed using high-sensitivity C-reactive protein (hs-CRP) and liver enzymes (Alanine Aminotransferase [ALT], Aspartate Aminotransferase [AST], Gamma-Glutamyl Transferase [GGT], and Alkaline Phosphatase [ALP]) as candidate mediators. while a two-step MR mediation analysis was implemented. First, the causal effects of depression on circulating C-Reactive Protein (CRP), GGT, and ALP were estimated; second, the causal effects of CRP, GGT, and ALP on MASLD were estimated. Genome-Wide Association Study (GWAS) sources included the GWAS Catalog entries GCST90310151 [14], ukb-d-30730 [15], and GCST90468060 [16].

Instrument construction proceeded by clumping single-nucleotide polymorphisms (SNPs) associated with the exposure at genome-wide significance (*p* < 5 × 10^−8^); when instruments were sparse, the threshold was relaxed to *p* < 1 × 10^−6^. Linkage disequilibrium–based clumping used r^2^ < 0.001 within a 10,000 kb window to retain independent variants.

Indirect Effects were computed using the product-of-coefficients method. The total effect corresponded to the direct MR estimate for depression to MASLD. The significance of the mediation effect was assessed with the use of parametric bootstrap with 5000 replicate samples (increased to 10,000 if a near null distribution was required) to construct robust confidence intervals. Sensitivity analyses included: (1) triangulation across inverse-variance weighting (IVW), weighted median, weighted mode, simple mode, and MR-Egger; (2) tests for heterogeneity (Cochran’s Q), horizontal pleiotropy (Egger intercept), and outliers (MR-PRESSO); and (3) leave-one-out analyses to detect influential SNPs, together with Steiger filtering to confirm causal direction.

### 2.4. Transcriptome Data Analysis

An integrated bioinformatics and machine-learning framework was applied to identify genes shared between MASLD and depression. Two public datasets were retrieved from the Gene Expression Omnibus (GEO): GSE240729 (MASLD) [17] and GSE98793 (depression) [18]. Weighted gene co-expression network analysis (WGCNA) was performed separately on female samples from each dataset to construct disease-specific co-expression networks. Modules correlated with the phenotype were identified and subjected to Gene Ontology (GO) enrichment analysis. Modules with the strongest phenotype association and highest functional coherence were retained for integration.

To extract the most discriminative features, genes from depression-associated modules were entered into a supervised-learning pipeline. Using these module genes as predictors, multiple classifiers—support vector machine (SVM), random forest (RF), Lasso-penalized logistic regression, and XGBoost—were trained to classify depression status. Performance was evaluated by the area under the receiver-operating characteristic curve (AUC-ROC). The top 20 features by importance from the best-performing model were intersected with genes from the key MASLD module to define robust shared genes across the two disorders.

### 2.5. Single-Cell Analysis

Single-cell RNA-sequencing data from 45 liver samples (GEO: GSE202379) were integrated, including healthy controls (*n* = 6), NAFLD (*n* = 7), nonalcoholic steatohepatitis (NASH; *n* = 28), and NASH cirrhosis (*n* = 4) [19]. Given that *CD4*^+^ T cells are the principal immune subset expressing *CD40LG* and that its binding partner *CD40* is predominantly expressed on B2 cells, lymphocytes and B2 cells were extracted for re-normalization, principal-component analysis, and clustering. *CD4*^+^ T cells were identified using a quantile-based dynamic threshold: a CD3 score was computed as the mean z-score of *CD3D*, *CD3E*, and *CD3G* expression; *CD4* expression was taken from the normalized count matrix. Cells exceeding both the 70th percentile of the CD3 score and the 70th percentile of *CD4* expression were classified as *CD4*^+^ T cells; remaining cells were labeled “other.” *CD40LG* expression in *CD4*^+^ T cells was compared across disease states, and *CD40* expression was assessed in B2 cells.

### 2.6. Statistical Analysis

All analyses were conducted in R 4.4.3 and STRING. Key packages included TwoSampleMR 0.6.22 and MRPRESSO 1.0 for Mendelian randomization; WGCNA 1.73 for network analysis; caret 7.0-1, randomForest 4.7-1.2, e1071 1.7-16, xgboost 1.7.11.1, and lightgbm 4.6.0 for machine learning; and Seurat 5.3.0/SeuratObject 5.2.0 for single-cell analyses.

## 3. Results

### 3.1. Association Between Depression and MASLD in Population-Based Data

**Figure 1:**
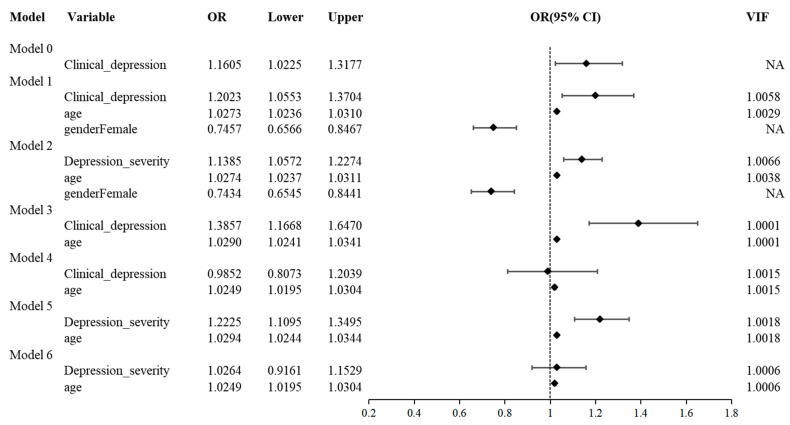
Multivariable logistic regression analysis of the association between depression and MASLD risk. This table summarizes a series of logistic regression models. Model 0 is unadjusted; Model 1 adjusts for age and sex; Model 2 treats depression severity as a continuous variable (also adjusting for age and sex). Models 3 and 4 are sex-stratified (females and males, respectively) adjusting for age; Models 5 and 6 repeat Models 3 and 4 with depression severity as the independent variable. Associations are expressed as odds ratios (ORs) with 95% confidence intervals (CIs).

Clinical depression was significantly associated with a higher risk of MASLD in unadjusted models (Model 0: OR = 1.16, 95% CI [1.02–1.32]). After adjusting for age and sex (Model 1), the association remained significant (OR = 1.20, 95% CI [1.06–1.37]). Treating depression severity as a continuous variable (Model 2) indicated that each incremental increase in severity grade corresponded to a 14% higher likelihood of MASLD (OR = 1.14, 95% CI [1.06–1.23]). All variance inflation factors were below 10, suggesting no multicollinearity (Figure 1).

**Figure 2:**
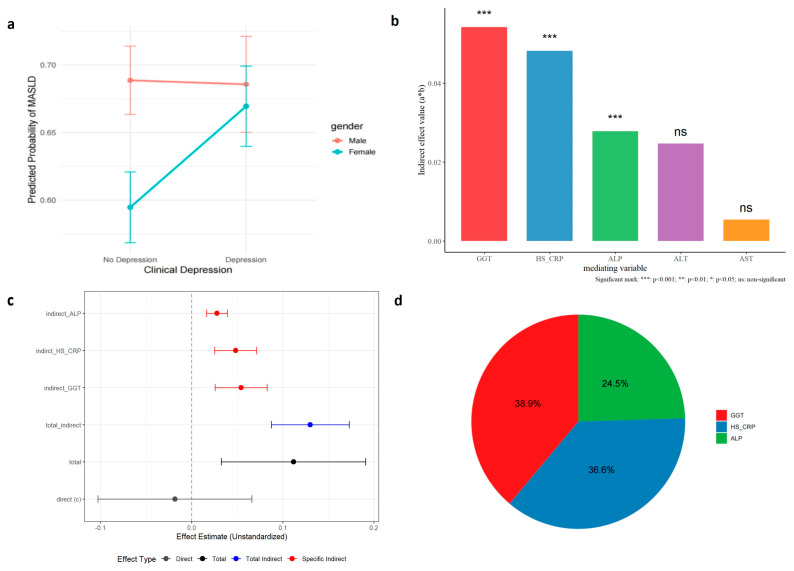
Full mediation of the depression-MASLD association through hs-CRP, GGT, and ALP. Observational mediation analysis of the association between depression and MASLD. (**a**) Single-mediator models demonstrating that hs-CRP, GGT, and ALP each have significant indirect effects when assessed individually. (**b**) In a parallel mediation model including all three mediators, the combined indirect effect remains highly significant (β = 0.13, *p* < 0.001), while the direct pathway from depression to MASLD becomes non-significant (β = −0.002, *p* = 0.73), indicating full mediation through inflammation and liver injury markers. The (a*b) on the vertical axis represents the mediating effect. That is, the product of the effect a of depression → GGT/HS_CRP/ALP/ALT/AST and the effect b of GGT/HS_CRP/ALP/ALT/AST → MASLD. (**c**) Forest plot summarizing the effect estimates for the mediation pathways. The direct effect of depression on MASLD is non-significant (crossing the zero line), whereas the total effect and total indirect effect are significantly positive. Specifically, the individual indirect effects mediated by ALP, hs-CRP, and GGT are all statistically significant, as indicated by their 95% confidence intervals being entirely above zero. (**d**) The proportion of mediating effects for the three significant mediating variables is shown. GGT had the strongest effect (38.9%). Hs_CRP followed with 36.6%. ALP was relatively weak at 24.5%.

Sex-stratified analyses revealed significant effect modification by sex. Among females (Model 3), depression was strongly associated with MASLD risk (OR = 1.39, 95% CI [1.17–1.65]), whereas no significant association was observed among males (Model 4: OR = 0.99, 95% CI [0.81–1.20]). Comparable results were seen when using depression severity as the independent variable (Model 5: OR = 1.22, 95% CI [1.11–1.35]; Model 6: OR = 1.03, 95% CI [0.92–1.15]). An interaction test confirmed the sex-dependent pattern (*p* = 0.01). As illustrated in Figure 2a, in males, the predicted probability of MASLD was essentially unchanged regardless of depression status (0.70 vs. 0.69). In women, however, depression was associated with an increase in predicted MASLD probability, from 0.59 (95% CI [0.55–0.63]) to 0.68 (95% CI [0.64–0.72]) (Supplementary Table S1).

### 3.2. Inflammatory and Hepatic Injury Biomarkers Mediate Involved in the Association

We evaluated the potential mediating roles of hepatic enzymes (ALT, AST, GGT, and ALP) and the systemic inflammatory marker hs-CRP in the relationship between depression and MASLD. Multiple mediation analyses indicated that GGT, hs-CRP, and ALP exerted significant mediating effects. When these significant mediators were simultaneously included in a parallel mediation model, the association between depression and MASLD became fully mediated by hs-CRP (representing systemic inflammation) and by GGT and ALP (reflecting hepatic injury), rendering the direct effect nonsignificant.

### 3.3. Genetic Evidence for a Causal Effect of Depression on MASLD

**Figure 3:**
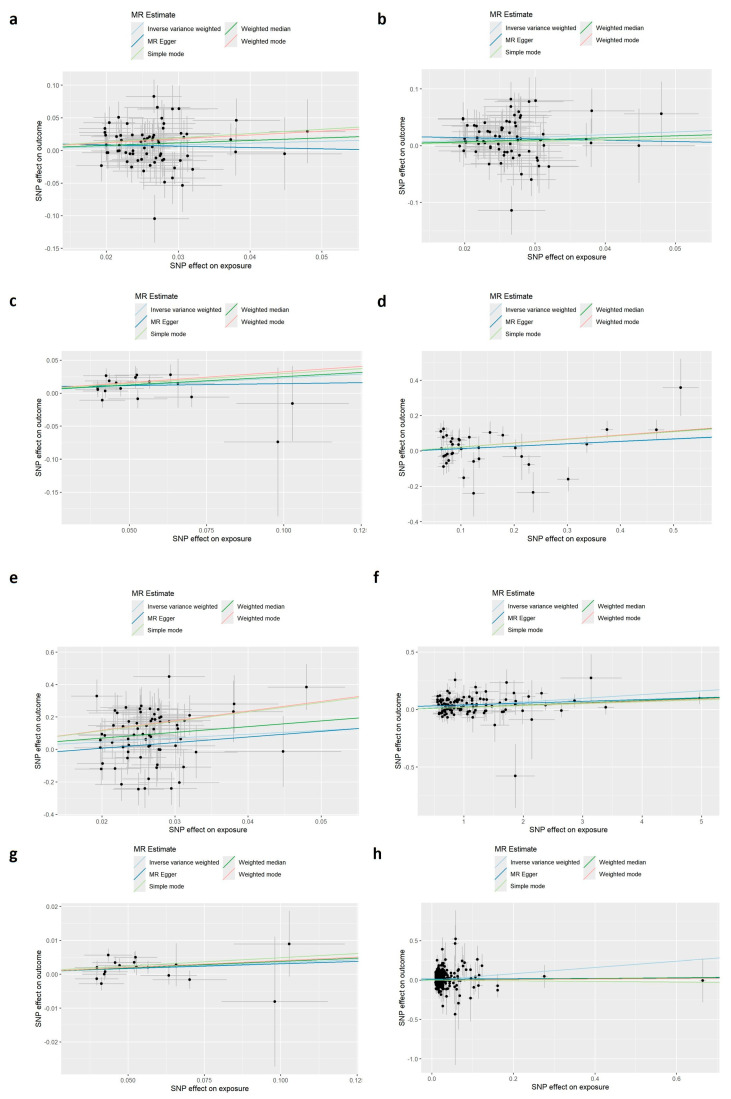
Causal effect of depression on MASLD and mediating roles of inflammation and liver injury markers. Mendelian randomization analysis of depression and MASLD. (**a**,**b**) Scatter plots of the main causal effect of depression on MASLD using FinnGen R12 (**a**) and FinnGen R10 (**b**) as outcomes. (**c**,**d**) Two-step MR for the depression → CRP → MASLD pathway: (**c**) effect of depression on CRP, (**d**) effect of CRP on MASLD. (**e**,**f**) Two-step MR for depression → GGT → MASLD: (**e**) effect of depression on GGT, (**f**) effect of GGT on MASLD. (**g**,**h**) Two-step MR for depression → ALP → MASLD: (**g**) effect of depression on ALP, (**h**) effect of ALP on MASLD.

To infer causality, we conducted two-sample MR analyses. Using the inverse variance–weighted (IVW) method, we observed a significant causal effect of genetically predicted depression on MASLD risk. This finding was consistently validated across two independent FinnGen cohorts—R10 (β = 0.483, SE = 0.146, *p* = 0.001) and R12 (β = 0.277, SE = 0.128, *p* = 0.031). The MR-Egger intercept test indicated no evidence of directional pleiotropy (*p* > 0.05), and MR-PRESSO analysis detected no outliers, supporting the robustness of the primary results (Figure 3a,b).

To explore potential mediation pathways between depression and MASLD, we used a two-step MR framework focusing on inflammation and liver injury markers. We separately evaluated systemic inflammation (CRP) and hepatic injury markers (GGT and ALP) as mediators. Two-step MR further confirmed that CRP (β_indirect = 0.008, *p* = 0.048), GGT (β_indirect = 0.095, *p* < 0.001), and ALP (β_indirect = 0.014, *p* = 0.010) jointly mediate the causal pathway, forming a genetic-level axis of depression → inflammation/liver injury → MASLD.

### 3.4. Transcriptomics Analysis Identified Immune-Inflammation Modules

**Figure 4:**
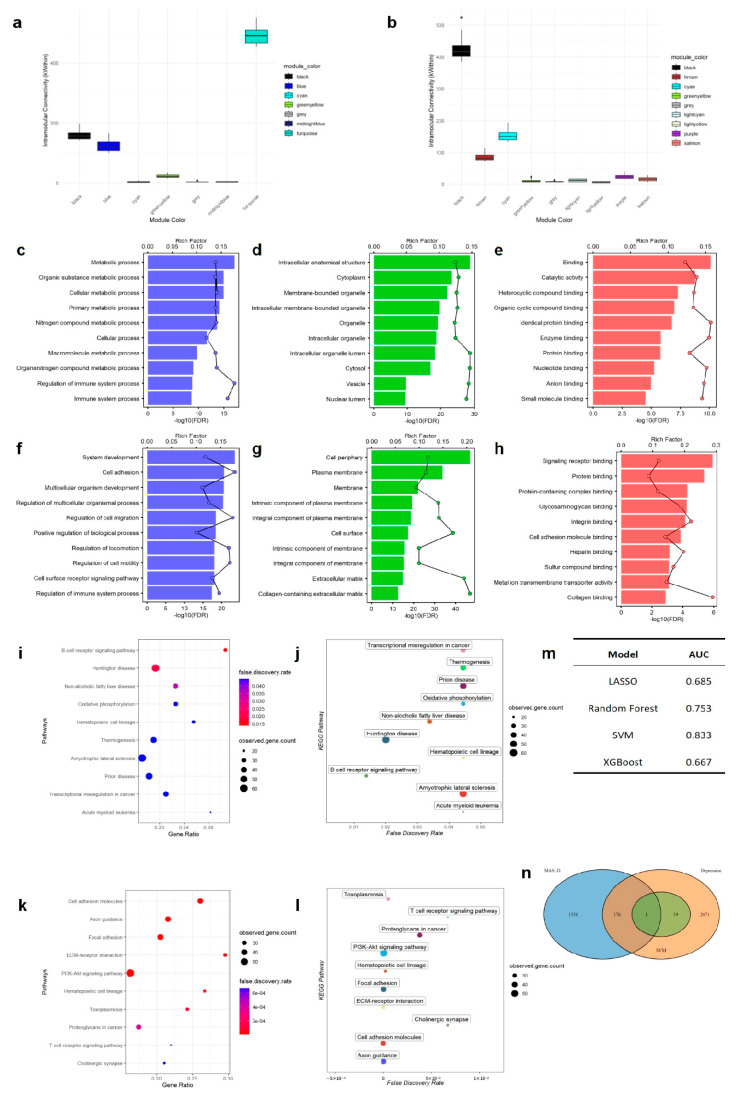
Transcriptomic analysis identifies shared immune-metabolic gene modules and nominates *CD40LG* as a key hub gene. (**a**,**b**) WGCNA results for the depression group (**a**) and MASLD group (**b**). The box plot shows the sum of within-module connectivity (kWithin) of the top 100 genes in each module. (**c**) Biological Process (BP) enrichment for the depression turquoise module. The top 10 enriched GO terms include “Metabolic process” and “Immune system process”. (**d**) Cellular Component (CC) enrichment for the depression turquoise module. GO terms highlight the “Intracellular anatomical structure” and “Cytoplasm”. (**e**) Molecular Function (MF) enrichment for the depression turquoise module. Predominant terms include “Binding,” “Catalytic activity,” and “Protein binding”. (**f**) Biological Process (BP) enrichment for the MASLD cyan module. High enrichment is seen in “System development” and “Regulation of immune system process”. (**g**) Cellular Component (CC) enrichment for the MASLD cyan module. Significant terms include “Cell periphery” and “Plasma membrane”. (**h**) Molecular Function (MF) enrichment for the MASLD cyan module. Terms include “Signaling receptor binding” and “Integrin binding”. (**i**) KEGG pathway bubble plot for the depression turquoise module. Pathways such as “B cell receptor signaling” and “Non-alcoholic fatty liver disease” are significantly enriched. (**j**) KEGG pathway FDR plot for the depression turquoise module. This illustrates the false discovery rate for pathways including “Oxidative phosphorylation” and “Thermogenesis”. (**k**) KEGG pathway bubble plot for the MASLD cyan module. Immune–metabolic crosstalk is indicated by enrichment in “PI3K-Akt signaling” and “T cell receptor signaling”. (**l**) KEGG pathway FDR plot for the MASLD cyan module. Terms such as “Focal adhesion” and “ECM–receptor interaction” show high significance. (**m**) Performance of machine learning models (LASSO, Random Forest, SVM, XGBoost) for hub gene prediction; SVM achieved the highest accuracy (AUC = 0.833). (**n**) Venn diagram showing overlapping hub genes between depression and MASLD identified by SVM; CD40LG was the only shared hub gene linking their immune–inflammatory pathways.

Weighted gene co-expression network analysis (WGCNA) identified co-expression modules closely associated with immune and inflammatory responses in both depression and MASLD datasets—the turquoise module in depression and the cyan module in MASLD. (Figure 4a,b) Functional enrichment analysis revealed that genes in these top modules were significantly enriched for GO terms related to immune system processes (Figure 4c–h). KEGG pathway analysis revealed that the depression-associated turquoise module was enriched for B cell signaling and the NAFLD pathway, while the MASLD-associated cyan module involved T cell signaling (Figure 4i–l), collectively highlighting adaptive immunity as a potential shared mechanism.

Using feature genes derived from WGCNA, multiple machine learning models were constructed to identify potential biomarkers. The support vector machine (SVM) algorithm demonstrated the best predictive performance (AUC = 0.833) (Figure 4m). By intersecting the top 20 important genes from the SVM models of both conditions, we identified one common hub gene—*CD40LG* (Figure 4n).

### 3.5. Single-Cell Analysis: Upregulation of CD40LG in Intrahepatic T Cells During Disease Progression

A total of 5755 lymphocytes were extracted from the single-cell dataset, including 3600 cells from male samples and 2155 from female samples. Among them, 243 B2 cells were identified (142 male, 101 female). Using a quantile-based thresholding approach, screening thresholds were set at the 70th percentile for CD3 (score = 0.266) and *CD4* (score = 0). Based on these criteria, 246 *CD4*^+^ T cells were identified, accounting for 4.3% of the total lymphocyte population.

**Figure 5:**
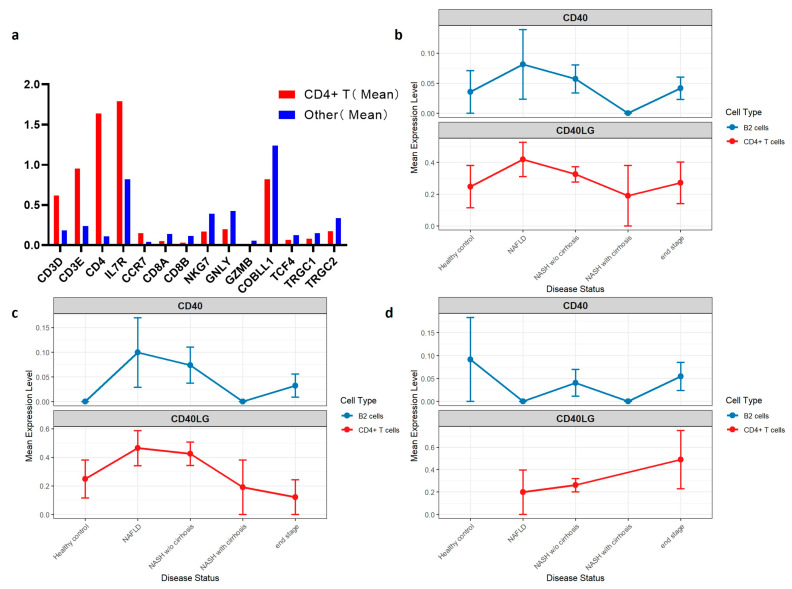
Integrative multi-omics identifies shared immune-metabolic gene modules and nominates *CD40LG* as a key hub gene. (**a**) Comparison of marker gene expression. This bar chart compares the mean expression levels of key markers between the identified *CD4*^+^ T cell population (red) and other cells (blue). The *CD4*^+^ T cell population shows significantly higher transcript levels of *CD3D*, *CD3E*, and *CD4*. (**b**) Dynamic expression of *CD40* and *CD40LG* in the total sample. The line plots illustrate the mean expression levels across disease states from healthy controls to end-stage liver disease. A peak in both *CD40* (in B2 cells) and *CD40LG* (in *CD4*^+^ T cells) is observed at the NAFLD/NASH stages. (**c**) Sex-stratified dynamic expression (Male). In male participants, the expression of *CD40* shows a marked peak at the NAFLD stage followed by a decline in cirrhosis. (**d**) Sex-stratified dynamic expression (Female). In female participants, while *CD40* expression shows a peak at the healthy control and NASH stages, *CD40LG* shows a progressive upward trend toward end-stage disease, highlighting potential sex-specific immune regulation.

The identified *CD4*^+^ T cells exhibited expected T cell marker gene expression patterns: canonical T cell markers were markedly elevated compared to other cells, including *CD4* (14.5-fold), *CD3E* (4.0-fold), *CD3D* (3.3-fold), *IL7R* (2.2-fold), and *CCR7* (3.7-fold). Expression of markers for other lymphocyte subtypes was very low in this population, confirming the specificity of the identification. These low markers included: *CD8A* (0.35-fold) and *CD8B* (0.26-fold) for CD8+ T cells; *NKG7* (0.43-fold) and *GNLY* (0.47-fold) for natural killer (NK) cells; *GZMB* (0.27-fold), *COBLL1* (0.64-fold), and *TCF4* (0.67-fold) for plasmacytoid dendritic (pDC) cells; and *TRGC1* (0.58-fold) and *TRGC2* (0.50-fold) for γδ T cells (Figure 5a, Supplementary Tables S2 and S3).

Expression analysis showed that *CD40LG* expression in *CD4*^+^ T cells peaked at the NAFLD stage (mean = 0.452), then declined during NASH without cirrhosis (mean = 0.321), and remained relatively low and stable in late stages (NASH with cirrhosis and end-stage disease, mean ≈ 0.310). Similarly, *CD40* expression in B2 cells peaked at NAFLD (mean = 0.008) before gradually declining. Due to small sample sizes, *CD40* expression was undetectable in NASH with cirrhosis (*n* = 3) and remained lower in end-stage disease (mean = 0.04) than in earlier stages (Figure 5b).

Sex-stratified analyses further revealed distinct trends. In males, *CD40LG* and *CD40* expression followed patterns similar to the overall cohort. In females, however, *CD40LG* expression in *CD4*^+^ T cells increased progressively with disease stage, while *CD40* expression in B2 cells showed no consistent pattern, likely due to limited sample size (Figure 5c,d) (see Supplementary Tables S4 and S5).

## 4. Discussion

Our study integrates multi-level evidence from epidemiology, genetics, and transcriptomics to investigate the association between depression and MASLD. We established a causal link from depression to MASLD and identified immune-inflammatory pathways, as a key underlying mechanism, with a notably stronger effect in women.

Epidemiological analysis of NHANES data revealed a clear association between depression and increased MASLD risk, with a marked sex difference: depression was associated with elevated MASLD risk in women but not in men. Mediation analysis identified inflammatory and liver injury markers—hs-CRP (indirect effect = 0.048, *p* < 0.001), GGT (indirect effect = 0.054, *p* < 0.001), and ALP (indirect effect = 0.028, *p* < 0.001)—as complete mediators of the depression–MASLD relationship. These findings suggest that systemic inflammation may link depression to hepatic steatosis and metabolic dysfunction.

To clarify causal direction, we performed bidirectional two-sample MR. Genetically predicted depression was associated with increased MASLD risk (R10: OR = 1.621, 95% CI [1.217–2.160]; R12: OR = 1.319, 95% CI [1.026–1.697]), whereas reverse-direction analysis did not support an effect of MASLD on depression. Sensitivity analyses reinforced the robustness of these findings. Through mediated MR, we established a causal pathway from depression to MASLD via CRP, GGT, and ALP, providing genetic evidence for a unidirectional effect of depression on liver injury through inflammatory and metabolic mechanisms.

At the molecular level, WGCNA of female transcriptome samples showed that the top module in depression (turquoise) and a key module in MASLD (cyan) were both enriched for immune-related mechanisms, including immune regulation and cytokine signaling pathways. Machine learning approaches then identified *CD40LG* as a shared core immune regulatory molecule. *CD40LG* is a co-stimulatory molecule on T cells that can activate antigen-presenting cells (e.g., B cells, dendritic cells, and macrophages) and stimulate the release of inflammatory cytokines by binding to *CD40* on these cells. The *CD40LG*–*CD40* axis may act as an immune transducer, mechanistically linking depression-associated central immune activation to peripheral hepatic inflammation and metabolic dysfunction.

To further validate and elucidate the role of *CD40LG*, we analyzed its expression and that of its ligand *CD40* across MASLD progression. In the overall sample, *CD40LG* in *CD4*^+^ T cells and *CD40* in B2 cells exhibited stage-dependent activation, peaking at the MASLD stage. The upregulation of *CD40LG* in *CD4*^+^ T cells and *CD40* in B2 cells suggests enhanced T cell–B cell interaction and an active adaptive immune response during the NAFLD stage. Similar patterns were observed in males. However, in females, *CD40LG* expression in *CD4*^+^ T cells increased progressively with disease stage, whereas *CD40* expression in B2 cells showed no consistent trend, possibly due to limited sample size. The different trend of *CD40LG* between males and females may underlie the stronger depression–MASLD association in women. Overall, *CD40LG* has potential as an immunological biomarker for the inflammatory transition phase of NAFLD and as a target for future immunomodulatory interventions.

Given that *CD40LG* is located on the X chromosome, we consider the sex-specific regulatory mechanisms, such as escape from X-chromosome inactivation or hormonal modulation, could predispose females to stronger *CD40LG*-mediated immune signaling. In males, any *CD40LG* variant is hemizygous (only one X chromosome), while females can be homozygous or heterozygous. However, due to X chromosome inactivation (XCI) in somatic cells, typically only one X allele is active in females to balance expression. A study on *CD40LG* polymorphisms in Kawasaki disease analyzed eight loci and found only small, non-significant differences in allele frequencies between sexes [20], suggesting that for common variants the male–female difference in “*CD40LG* positivity” is likely minimal. Some literature indicates that escape from XCI may vary by cell type, but there is no consensus that *CD40LG* is a conventional XCI-escape gene [21]. We propose that sex differences in *CD40LG* expression or XCI escape may play a role, but this requires direct validation (e.g., allele-specific single-cell expression, RNA-FISH, or single-cell eQTL analyses).

Our findings suggest that *CD40LG* is not merely a marker of immune activation in MASLD, but may be involved in the critical transition from reversible metabolic impairment to irreversible inflammatory fibrosis, potentially mediating sex disparities. However, due to the limited number of cells, this conclusion merits further investigation in larger, sex-balanced cohorts. Future studies should validate *CD40LG*’s functional role in the liver microenvironment and explore its therapeutic potential as an immunomodulatory target, including its effects on sex-specific differences.

Furthermore, beyond genetic factors, sex hormones and neuroendocrine pathways may contribute to the observed sex disparity. Estrogen exhibits immunomodulatory properties that could influence inflammatory tone and adaptive immune responses, potentially altering *CD40LG*–*CD40* signaling. Depression-associated dysregulation of the hypothalamic–pituitary–adrenal (HPA) axis and elevated cortisol may further promote hepatic inflammation and metabolic dysfunction [22]. These hormonal influences, together with X-linked genetic regulation, likely interact to shape the stronger depression–MASLD association observed in women.

The primary finding of our study—a unidirectional causal effect of depression on MASLD—is supported by several Mendelian randomization studies [9,23,24], strengthening the genetic evidence for depression as an upstream risk factor. The marked sex-specific association we observed, with effects concentrated in women, also aligns with epidemiological findings across diverse populations [9,25]. More importantly, our multi-omics approach provides mechanistic granularity: the female-predominant transcriptomic signature and the progressive upregulation of *CD40LG* in intrahepatic *CD4*^+^ T cells offer a plausible molecular substrate for this epidemiological and genetic signal. Although some prospective studies have suggested that baseline NAFLD may increase subsequent depression risk [26,27]—a finding not corroborated by our reverse MR analysis—the discrepancy between observational and genetic evidence may reflect methodological limitations and stage-dependent effects. In advanced disease stages, hepatic inflammation and psychosocial burden could jointly contribute to emotional disorders, forming a potential bidirectional cycle.

Our findings have important implications for clinical practice and public health. First, psychiatrists and hepatologists should be vigilant about comorbidity risks, especially in female patients with depression who also have metabolic syndrome or elevated inflammatory markers. It may be advisable to incorporate liver enzyme testing, hs-CRP measurement, and liver imaging into the routine management of these patients. Second, the blood biomarker *CD40LG* identified in this study holds promise for developing biomarker panels to identify high-risk individuals and enable targeted interventions. Third, our single-cell analysis suggests a potential intervention window early in MASLD progression. The peak expression of *CD40LG* in *CD4*^+^ T cells during the NAFLD stage indicates that immune activation via this axis is prominent during initial metabolic steatosis, before advanced fibrosis develops. This raises the possibility that early, depression-targeted anti-inflammatory strategies—whether pharmacological or behavioral—could attenuate immune-mediated hepatic injury and prevent transition to irreversible fibrotic disease.

This study has several limitations. First, the cross-sectional nature of the NHANES data restricts temporal inference, and the definitions of both depression and MASLD partly rely on subjective questionnaires, which may introduce bias. Despite multivariate adjustment, residual confounding such as diet and medication was difficult to completely rule out. Second, the observational mediation analysis depends on assumptions—such as correct temporal ordering and the absence of unmeasured confounding—that are difficult to fully verify. Moreover, the inflammatory marker hs_CRP used in the analysis is not specific to depression and may reflect processes related to other conditions or downstream effects rather than serving as a distinct causal intermediary. Third, in the mediation MR analysis, while we ruled out horizontal pleiotropy through methods such as MR-Egger and MR-PRESSO, the possibility of vertical pleiotropy cannot be completely excluded. Depression may elevate CRP, ALP, and GGT through other indirect pathways. Additionally, the transcriptomic data for depression were derived from peripheral blood, which may introduce tissue-specific noise. Finally, the functional mechanisms and clinical relevance of the *CD40LG*–*CD40* axis, as well as the sex-specific patterns observed in the single-cell analysis, due to the limitation of single-cell data set, the stability of the results obtained is not strong, and further analysis is still needed in larger sample sizes and more balanced gender ratio data sets.

Building on our findings, several key research avenues emerge. First, prospective cohort studies measuring *CD40LG* (and related inflammatory markers) in depressed individuals are needed to validate its predictive value for MASLD development. Second, experimental models (e.g., chronic stress in hepatosteatosis models) should be used to mechanistically interrogate the role of the *CD40LG*-*CD40* axis in linking neuroinflammation to liver pathology. Third, our study underscores the need for clinical trials to evaluate whether anti-inflammatory interventions or *CD40* pathway modulation can improve metabolic outcomes in patients with comorbid depression and early-stage MASLD, particularly women. Finally, integrating metabolomic and lipidomic data in future multi-omics frameworks is crucial. Our genetic and transcriptomic evidence delineates a pathway from depression to MASLD; metabolomics can provide direct functional validation. For instance, NMR-based metabolomics reveals that depression is associated with a distinct serum profile—elevated triglycerides, VLDL, the inflammatory marker GlycA, and reduced glutamine—which is linked to MASLD-related metabolic dysfunction independently of liver disease severity [28]. This aligns with and functionally extends our inflammation-mediated hypothesis. Such integration could precisely map the downstream metabolic consequences of this immune-mediated pathway and identify clinically translatable biomarkers.

## 5. Conclusions

This study provides evidence that depression is a risk factor for MASLD, mediated substantially by inflammation and demonstrating a clear female preponderance. Furthermore, employing a multidisciplinary approach provides converging multi-omics evidence and generates a testable hypothesis regarding the *CD40LG*–*CD40* axis. Collectively, our results argue for integrating liver surveillance into depression care and for exploring immunomodulatory strategies to reduce the prevalence of depression combined with MASLD, improve the quality of life of patients, and reduce medical costs.

## Figures and Tables

**Table 1:** Baseline characteristics of the study population. Participants’ sociodemographic and clinical features are summarized by MASLD status. Categorical variables are presented as percentages. MASLD and depression were defined as described above.

Variable	Level	No MASLD (*n* = 1596)	MASLD (*n* = 2912)
Age group	<20	148 (9.3)	74 (2.5)
	20–39	676 (42.4)	751 (25.8)
	40–59	398 (24.9)	1068 (36.7)
	≥60	374 (23.4)	1019 (35.0)
Gender	Male	661 (41.4)	1384 (47.5)
	Female	935 (58.6)	1528 (52.5)
Race	Mexican American	139 (9.3)	410 (14.9)
	Other Hispanic	159 (10.6)	314 (11.4)
	Non-Hispanic White	583 (38.8)	1116 (40.7)
	Non-Hispanic Black	458 (30.5)	643 (23.4)
	Non-Hispanic Asian	163 (10.9)	260 (9.5)
	Other/Multi-Racial	0 (0.0)	0 (0.0)
Clinical depression	No	1031 (64.6)	1780 (61.1)
	Yes	565 (35.4)	1132 (38.9)

## Data Availability

All data supporting the findings of this study are publicly available. The epidemiological data were obtained from the National Health and Nutrition Examination Survey (https://wwwn.cdc.gov/nchs/nhanes/Default.aspx; accessed on 11 August 2025). Genetic association data for depression, CRP, liver enzymes, and MASLD were sourced from the GWAS Catalog (https://www.ebi.ac.uk/gwas/; accessed on 5 September 2025), the IEU OpenGWAS database (https://gwas.mrcieu.ac.uk/; accessed on 5 September 2025), and the FinnGen consortium. Transcriptomic datasets (including bulk and single-cell RNA-seq data) were downloaded from the Gene Expression Omnibus (https://www.ncbi.nlm.nih.gov/geo/summary/; accessed on 11 October 2025), with specific accession numbers provided in the Section 2 (Materials and Methods).

## Supplementary Materials

The following supporting information can be downloaded at: https://www.mdpi.com/article/10.3390/biomedicines14010174/s1, Supplementary Table S1. Multivariate logistic regression of MASLD risk. Supplementary Table S2. Mediation analysis of the depression-MASLD association. Supplementary Table S3. Evidence supporting the specific identification of *CD4*^+^ T cells. Supplementary Table S4. *CD40LG* expression in *CD4*^+^ T cells during MASLD progression. Supplementary Table S5. *CD40* expression in B2 cells during MASLD progression.

## Abbreviations

The following abbreviations are used in this manuscript:

ALP	Alkaline Phosphatase
ALT	Alanine Aminotransferase
AST	Aspartate Aminotransferase
AUC-ROC	Area Under the Receiver-Operating Characteristic Curve
BMI	Body-Mass Index
BP	Biological Process
CAP	Controlled Attenuation Parameter
CC	Cellular Component
CI	Confidence Interval
CRP	C-Reactive Protein
DALYs	Disability-Adjusted Life Years
eQTL	expression Quantitative Trait Loci
FDR	False Discovery Rate
GBD	Global Burden of Disease
GEO	Gene Expression Omnibus
GGT	Gamma-Glutamyl Transferase
GO	Gene Ontology
GWAS	Genome-Wide Association Study
HDL-C	High-Density Lipoprotein Cholesterol
hs-CRP	High-Sensitivity C-Reactive Protein
IVW	Inverse-Variance Weighting
KEGG	Kyoto Encyclopedia of Genes and Genomes
LASSO	Least Absolute Shrinkage and Selection Operator
MASLD	Metabolic Dysfunction–Associated Steatotic Liver Disease
MF	Molecular Function
MR	Mendelian Randomization
NAFLD	Nonalcoholic Fatty Liver Disease
NASH	Nonalcoholic Steatohepatitis
NHANES	National Health and Nutrition Examination Survey
OR	Odds Ratio
PHQ-9	9-item Patient Health Questionnaire
RF	Random Forest
ROC	Receiver Operating Characteristic
SE	Standard Error
SNPs	Single-Nucleotide Polymorphisms
SVM	Support Vector Machine
VIFs	Variance Inflation Factors
WGCNA	Weighted Gene Co-expression Network Analysis
XCI	X chromosome inactivation
XGBoost	Extreme Gradient Boosting
