# Supplementary material for: Multi-Omics Evidence Linking Depression to MASLD Risk via Inflammatory Immune Signaling
Source: Biomedicines. 2026 Jan 13;14(1):174. doi: 10.3390/biomedicines14010174 (PMC12839395; doi:10.3390/biomedicines14010174)
Supplement: Supplementary file 1 [file biomedicines-14-00174-s001.zip › biomedicines-4057959-supplementary.pdf]

Supplementary Materials:

Supplementary Table S1. Multivariate logistic regression of MASLD risk.

Association between clinical depression, gender, age, and MASLD risk. Results from a multivariate logistic regression model, adjusted for age, including a clinical depression-by-gender interaction term. The model reveals a significant interaction, with depression associated with increased MASLD risk in women but not in men.

term	estimate	SE	p	95%CI
(Intercept)	0.61	0.10	0.00	[0.50,0.75]
Clinical_depression	0.99	0.10	0.91	[0.81,1.21]
genderFemale	0.66	0.08	0.00	[0.56,0.77]
age	1.03	0.00	0.00	[1.02,1.03]
Clinical_depression:genderFemale	1.40	0.13	0.01	[1.08,1.82]

Supplementary Table S2. Mediation analysis of the depression-MASLD association.

Assessment of ALT, AST, GGT, ALP and Hs_CRP as mediators between depression and MASLD. The results from both single and parallel mediation analyses indicate that the relationship is completely mediated by GGT, ALP and Hs_CRP, with no significant direct effect observed after accounting for their combined indirect pathways.

**Mediator**	**Single Mediator Analysis**		**Parallel Mediation Analysis**	
	**Indirect Effect (P-value)**	**Direct Effect (P-value)**	**Indirect Effect (P-value)**	**Std. Error**
ALT	0.025 (0.058)	0.087 (0.029)	-	-
AST	0.005 (0.147)	0.106 (0.008)	-	-
GGT	0.054 (<0.001)	0.057 (0.165)	0.05 (<0.001)	0.01
ALP	0.028 (<0.001)	0.084 (0.038)	0.03 (<0.001)	0.01
Hs_CRP	0.048 (<0.001)	0.064 (0.122)	0.05 (<0.001)	0.01
Total Indirect Effect	-	-	0.13 (<0.001)	0.02
Total Effect (c)	-	-	0.11 (0.01)	0.04
Direct Effect (c')	-	-	-0.02 (0.68)	0.04

Supplementary Table S3. Evidence supporting the specific identification of CD4+ T cells.

Validation of CD4+ T cell identification from liver Lymphocytes. This table compares the average expression levels of key lineage marker genes between the identified CD4+ T cells and all other cells. High expression of CD4+ T cell markers (CD4, CD3E, CD3D, IL7R, CCR7) coupled with low expression

of markers for other lymphocyte populations (CD8+ T, NK, pDC, and γδ T cells) confirms the specific and successful isolation of CD4+ T cells for downstream analysis.

Gene	CD4+ T（Mean）	Other（Mean）	Foldchange
CD3D	0.618	0.185	3.338
CD3E	0.953	0.240	3.968
CD4	1.639	0.113	14.456
IL7R	1.791	0.822	2.179
CCR7	0.151	0.040	3.750
CD8A	0.051	0.143	0.354
CD8B	0.030	0.117	0.259
NKG7	0.167	0.391	0.428
GNLY	0.201	0.427	0.472
GZMB	0.019	0.059	0.329
COBLL1	0.822	1.239	0.664
TCF4	0.067	0.127	0.531
TRGC1	0.083	0.152	0.543
TRGC2	0.173	0.337	0.513

Supplementary Table S4. CD40LG expression in CD4+ T cells during MASLD progression.

CD40LG expression peaks during the NAFLD stage and declines in subsequent stages (NASH without cirrhosis, NASH with cirrhosis, and end-stage) in the overall cohort and in males. In contrast, CD40LG expression in females shows a distinct pattern, increasing progressively with disease advancement.

Celltype	Disease.status	mean_expr	sd_expr	n_cells	prop_positive
All sex					
CD4+T cell	Healthy control	0.20	0.47	19	0.16
CD4+T cell	NAFLD	0.45	0.66	42	0.33
CD4+T cell	NASH w/o cirrhosis	0.32	0.61	198	0.23
CD4+T cell	NASH with cirrhosis	0.31	0.58	8	0.25
CD4+T cell	end stage	0.31	0.68	32	0.19
Other cell	Healthy control	0.06	0.31	595	0.04
Other cell	NAFLD	0.07	0.33	688	0.04
Other cell	NASH w/o cirrhosis	0.08	0.36	2767	0.05
Other cell	NASH with cirrhosis	0.07	0.32	165	0.05
Other cell	end stage	0.06	0.31	1241	0.03
Male					
CD4+T cell	Healthy control	0.20	0.47	19	0.16
CD4+T cell	NAFLD	0.51	0.69	35	0.37
CD4+T cell	NASH w/o cirrhosis	0.40	0.64	83	0.29
CD4+T cell	NASH with cirrhosis	0.31	0.58	8	0.25
CD4+T cell	end stage	0.17	0.52	19	0.11
Other cell	Healthy control	0.06	0.32	551	0.04
Other cell	NAFLD	0.06	0.33	526	0.04
Other cell	NASH w/o cirrhosis	0.07	0.33	1346	0.05
Other cell	NASH with cirrhosis	0.08	0.34	141	0.06
Other cell	end stage	0.05	0.28	872	0.03
Female					
CD4+T cell	Healthy control	0.20	0.47	19	0.16
CD4+T cell	NAFLD	0.51	0.69	35	0.37
CD4+T cell	NASH w/o cirrhosis	0.40	0.64	83	0.29
CD4+T cell	NASH with cirrhosis	0.31	0.58	8	0.25
CD4+T cell	end stage	0.17	0.52	19	0.11
Other cell	Healthy control	0.06	0.32	551	0.04
Other cell	NAFLD	0.06	0.33	526	0.04
Other cell	NASH w/o cirrhosis	0.07	0.33	1346	0.05
Other cell	NASH with cirrhosis	0.08	0.34	141	0.06
Other cell	end stage	0.05	0.28	872	0.03

Supplementary Table S5. CD40 expression in B2 cells during MASLD progression.

CD40 expression levels in B2 cells of different MASLD stages and with sex stratification. Its expression trend was similar to that of its ligand (CD40LG) in the whole cohort and in men, showing a peak followed by a decrease in NAFLD. Interpretation of expression trends in females was limited by the small sample size that did not allow stable expression patterns to be identified in the sex-stratified analysis.

Disease.status	mean_expr	sd_expr	n_cells	prop_positive
All gender				
Healthy control	0.04	0.20	31	0.03
NAFLD	0.08	0.27	22	0.09
NASH w/o cirrhosis	0.06	0.22	87	0.07
NASH with cirrhosis	0.00	0.00	10	0.00
end stage	0.04	0.18	93	0.05
Male				
Healthy control	0.00	0.00	19	0.00
NAFLD	0.10	0.30	18	0.11
NASH w/o cirrhosis	0.07	0.24	43	0.09
NASH with cirrhosis	0.00	0.00	7	0.00
end stage	0.03	0.17	55	0.04
Female				
Healthy control	0.09	0.32	12	0.08
NAFLD	0.00	0.00	4	0.00
NASH w/o cirrhosis	0.04	0.19	44	0.05
NASH with cirrhosis	0.00	0.00	3	0.00
end stage	0.05	0.19	38	0.08
